# Supplementary material for: Understanding the Potential Gene Regulatory Network of Starch Biosynthesis in Tartary Buckwheat by RNA-Seq
Source: Int J Mol Sci. 2022 Dec 12;23(24):15774. doi: 10.3390/ijms232415774 (PMC9779217; doi:10.3390/ijms232415774)
Supplement: Supplementary file 1 [file ijms-23-15774-s001.zip › Supplementary Figure S2. Top 20 enriched KEGG pathways of the transcriptome.pdf]

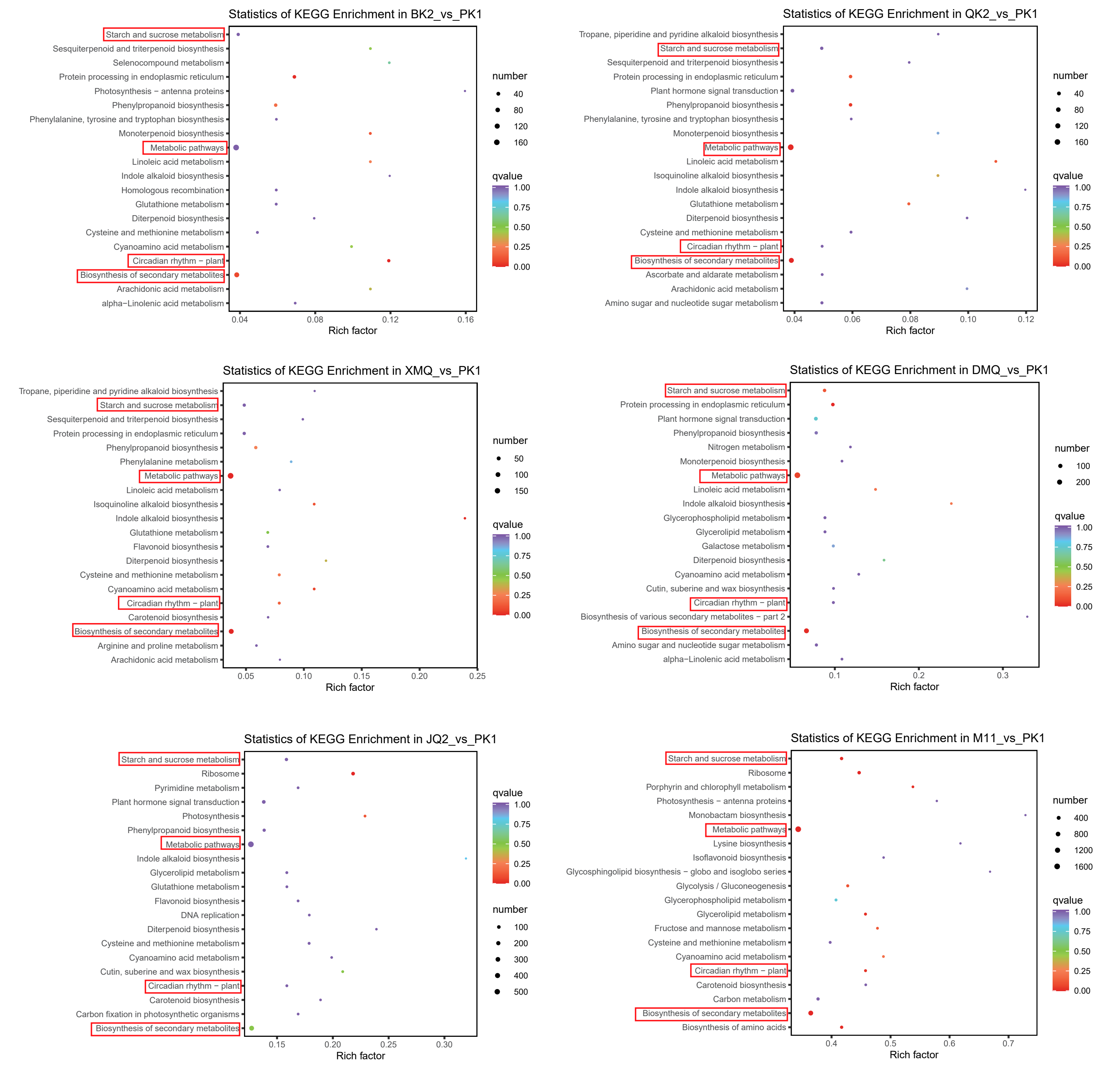
Statistics of KEGG Enrichment in BK2_vs_PK1
Starch and sucrose metabolism
Sesquiterpenoid and triterpenoid biosynthesis
Selenocompound metabolism
Protein processing in endoplasmic reticulum
Photosynthesis − antenna proteins
Phenylpropanoid biosynthesis
Phenylalanine, tyrosine and tryptophan biosynthesis
Monoterpenoid biosynthesis
Metabolic pathways
Linoleic acid metabolism
Indole alkaloid biosynthesis
Homologous recombination
Glutathione metabolism
Diterpenoid biosynthesis
Cysteine and methionine metabolism
Cyanoamino acid metabolism
Circadian rhythm − plant
Biosynthesis of secondary metabolites
Arachidonic acid metabolism
alpha−Linolenic acid metabolism
0.04
0.08
0.12
0.16
Rich factor
number
40
80
120
160
qvalue
1.00
0.75
0.50
0.25
0.00
Statistics of KEGG Enrichment in QK2_vs_PK1
Tropane, piperidine and pyridine alkaloid biosynthesis
Starch and sucrose metabolism
Sesquiterpenoid and triterpenoid biosynthesis
Protein processing in endoplasmic reticulum
Plant hormone signal transduction
Phenylpropanoid biosynthesis
Phenylalanine, tyrosine and tryptophan biosynthesis
Monoterpenoid biosynthesis
Metabolic pathways
Linoleic acid metabolism
Isoquinoline alkaloid biosynthesis
Indole alkaloid biosynthesis
Glutathione metabolism
Diterpenoid biosynthesis
Cysteine and methionine metabolism
Circadian rhythm − plant
Biosynthesis of secondary metabolites
Ascorbate and aldarate metabolism
Arachidonic acid metabolism
Amino sugar and nucleotide sugar metabolism
0.04
0.06
0.08
0.10
0.12
Rich factor
number
40
80
120
160
qvalue
1.00
0.75
0.50
0.25
0.00
Statistics of KEGG Enrichment in XMQ_vs_PK1
Tropane, piperidine and pyridine alkaloid biosynthesis
Starch and sucrose metabolism
Sesquiterpenoid and triterpenoid biosynthesis
Protein processing in endoplasmic reticulum
Phenylpropanoid biosynthesis
Phenylalanine metabolism
Metabolic pathways
Linoleic acid metabolism
Isoquinoline alkaloid biosynthesis
Indole alkaloid biosynthesis
Glutathione metabolism
Flavonoid biosynthesis
Diterpenoid biosynthesis
Cysteine and methionine metabolism
Cyanoamino acid metabolism
Circadian rhythm − plant
Carotenoid biosynthesis
Biosynthesis of secondary metabolites
Arginine and proline metabolism
Arachidonic acid metabolism
0.05
0.10
0.15
0.20
0.25
Rich factor
number
50
100
150
qvalue
1.00
0.75
0.50
0.25
0.00
Statistics of KEGG Enrichment in DMQ_vs_PK1
Starch and sucrose metabolism
Protein processing in endoplasmic reticulum
Plant hormone signal transduction
Phenylpropanoid biosynthesis
Nitrogen metabolism
Monoterpenoid biosynthesis
Metabolic pathways
Linoleic acid metabolism
Indole alkaloid biosynthesis
Glycerophospholipid metabolism
Glycerolipid metabolism
Galactose metabolism
Diterpenoid biosynthesis
Cyanoamino acid metabolism
Cutin, suberine and wax biosynthesis
Circadian rhythm − plant
Biosynthesis of various secondary metabolites − part 2
Biosynthesis of secondary metabolites
Amino sugar and nucleotide sugar metabolism
alpha−Linolenic acid metabolism
0.1
0.2
0.3
Rich factor
number
100
200
qvalue
1.00
0.75
0.50
0.25
0.00
Statistics of KEGG Enrichment in JQ2_vs_PK1
Starch and sucrose metabolism
Ribosome
Pyrimidine metabolism
Plant hormone signal transduction
Photosynthesis
Phenylpropanoid biosynthesis
Metabolic pathways
Indole alkaloid biosynthesis
Glycerolipid metabolism
Glutathione metabolism
Flavonoid biosynthesis
DNA replication
Diterpenoid biosynthesis
Cysteine and methionine metabolism
Cyanoamino acid metabolism
Cutin, suberine and wax biosynthesis
Circadian rhythm − plant
Carotenoid biosynthesis
Carbon fixation in photosynthetic organisms
Biosynthesis of secondary metabolites
0.15
0.20
0.25
0.30
Rich factor
qvalue
1.00
0.75
0.50
0.25
0.00
number
100
200
300
400
500
Statistics of KEGG Enrichment in M11_vs_PK1
Starch and sucrose metabolism
Ribosome
Porphyrin and chlorophyll metabolism
Photosynthesis − antenna proteins
Monobactam biosynthesis
Metabolic pathways
Lysine biosynthesis
Isoflavonoid biosynthesis
Glycosphingolipid biosynthesis − globo and isoglobo series
Glycolysis / Gluconeogenesis
Glycerophospholipid metabolism
Glycerolipid metabolism
Fructose and mannose metabolism
Cysteine and methionine metabolism
Cyanoamino acid metabolism
Circadian rhythm − plant
Carotenoid biosynthesis
Carbon metabolism
Biosynthesis of secondary metabolites
Biosynthesis of amino acids
0.4
0.5
0.6
0.7
Rich factor
number
400
800
1200
1600
qvalue
1.00
0.75
0.50
0.25
0.00
