# Supplementary material for: Understanding the Potential Gene Regulatory Network of Starch Biosynthesis in Tartary Buckwheat by RNA-Seq
Source: Int J Mol Sci. 2022 Dec 12;23(24):15774. doi: 10.3390/ijms232415774 (PMC9779217; doi:10.3390/ijms232415774)
Supplement: Supplementary file 1 [file ijms-23-15774-s001.zip › Supplementary Figure S4. Top 50 enriched GO items of the transcriptome.pdf]

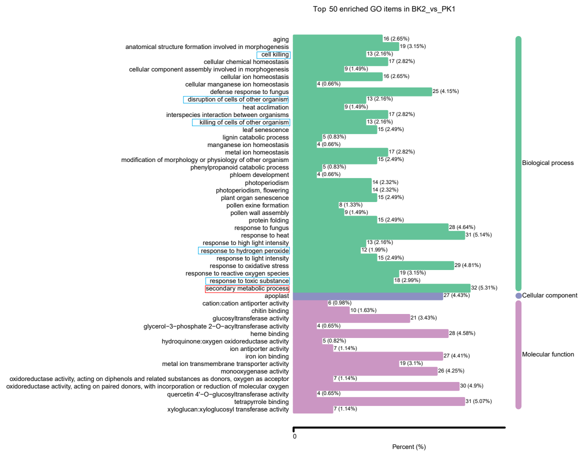

Top 50 enriched GO items in BK2_vs_PK1
aging 16 (2.65%)
anatomical structure formation involved in morphogenesis 19 (3.15%)
cell killing 13 (2.16%)
cellular chemical homeostasis 17 (2.82%)
cellular component assembly involved in morphogenesis 9 (1.49%)
cellular ion homeostasis 16 (2.65%)
cellular manganese ion homeostasis 4 (0.66%)
defense response to fungus 25 (4.15%)
disruption of cells of other organism 13 (2.16%)
heat acclimation 9 (1.49%)
interspecies interaction between organisms 17 (2.82%)
killing of cells of other organism 13 (2.16%)
leaf senescence 15 (2.49%)
lignin catabolic process 5 (0.83%)
manganese ion homeostasis 4 (0.66%)
metal ion homeostasis 17 (2.82%)
modification of morphology or physiology of other organism 15 (2.49%)
phenylpropanoid catabolic process 5 (0.83%)
phloem development 4 (0.66%)
photoperiodism 14 (2.32%)
photoperiodism, flowering 14 (2.32%)
plant organ senescence 15 (2.49%)
pollen exine formation 8 (1.33%)
pollen wall assembly 9 (1.49%)
protein folding 15 (2.49%)
response to fungus 28 (4.64%)
response to heat 31 (5.14%)
response to high light intensity 13 (2.16%)
response to hydrogen peroxide 12 (1.99%)
response to light intensity 15 (2.49%)
response to oxidative stress 29 (4.81%)
response to reactive oxygen species 19 (3.15%)
response to toxic substance 18 (2.99%)
secondary metabolic process 32 (5.31%)
apoplast 27 (4.43%)
cation:cation antiporter activity 6 (0.98%)
chitin binding 10 (1.63%)
glucosyltransferase activity 21 (3.43%)
glycerol−3−phosphate 2−O−acyltransferase activity 4 (0.65%)
heme binding 28 (4.58%)
hydroquinone:oxygen oxidoreductase activity 5 (0.82%)
ion antiporter activity 7 (1.14%)
iron ion binding 27 (4.41%)
metal ion transmembrane transporter activity 19 (3.1%)
monooxygenase activity 26 (4.25%)
oxidoreductase activity, acting on diphenols and related substances as donors, oxygen as acceptor 7 (1.14%)
oxidoreductase activity, acting on paired donors, with incorporation or reduction of molecular oxygen 30 (4.9%)
quercetin 4'−O−glucosyltransferase activity 4 (0.65%)
tetrapyrrole binding 31 (5.07%)
xyloglucan:xyloglucosyl transferase activity 7 (1.14%)
Biological process
Cellular component
Molecular function
0
Percent (%)

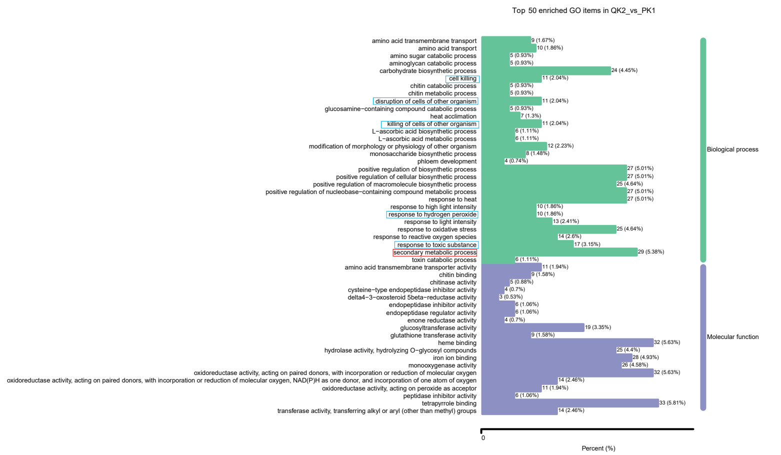

Top 50 enriched GO items in QK2_vs_PK1
amino acid transmembrane transport 9 (1.67%)
amino acid transport 10 (1.86%)
amino sugar catabolic process 5 (0.93%)
aminoglycan catabolic process 5 (0.93%)
carbohydrate biosynthetic process 24 (4.45%)
cell killing 11 (2.04%)
chitin catabolic process 5 (0.93%)
chitin metabolic process 5 (0.93%)
disruption of cells of other organism 11 (2.04%)
glucosamine−containing compound catabolic process 5 (0.93%)
heat acclimation 7 (1.3%)
killing of cells of other organism 11 (2.04%)
L−ascorbic acid biosynthetic process 6 (1.11%)
L−ascorbic acid metabolic process 6 (1.11%)
modification of morphology or physiology of other organism 12 (2.23%)
monosaccharide biosynthetic process 8 (1.48%)
phloem development 4 (0.74%)
positive regulation of biosynthetic process 27 (5.01%)
positive regulation of cellular biosynthetic process 27 (5.01%)
positive regulation of macromolecule biosynthetic process 25 (4.64%)
positive regulation of nucleobase−containing compound metabolic process 27 (5.01%)
response to heat 27 (5.01%)
response to high light intensity 10 (1.86%)
response to hydrogen peroxide 10 (1.86%)
response to light intensity 13 (2.41%)
response to oxidative stress 25 (4.64%)
response to reactive oxygen species 14 (2.6%)
response to toxic substance 17 (3.15%)
secondary metabolic process 29 (5.38%)
toxin catabolic process 6 (1.11%)
amino acid transmembrane transporter activity 11 (1.94%)
chitin binding 9 (1.58%)
chitinase activity 5 (0.88%)
cysteine−type endopeptidase inhibitor activity 4 (0.7%)
delta4−3−oxosteroid 5beta−reductase activity 3 (0.53%)
endopeptidase inhibitor activity 6 (1.06%)
endopeptidase regulator activity 6 (1.06%)
enone reductase activity 4 (0.7%)
glucosyltransferase activity 19 (3.35%)
glutathione transferase activity 9 (1.58%)
heme binding 32 (5.63%)
hydrolase activity, hydrolyzing O−glycosyl compounds 25 (4.4%)
iron ion binding 28 (4.93%)
monooxygenase activity 26 (4.58%)
oxidoreductase activity, acting on paired donors, with incorporation or reduction of molecular oxygen 32 (5.63%)
oxidoreductase activity, acting on paired donors, with incorporation or reduction of molecular oxygen, NAD(P)H as one donor, and incorporation of one atom of oxygen 14 (2.46%)
oxidoreductase activity, acting on peroxide as acceptor 11 (1.94%)
peptidase inhibitor activity 6 (1.06%)
tetrapyrrole binding 33 (5.81%)
transferase activity, transferring alkyl or aryl (other than methyl) groups 14 (2.46%)
Biological process
Molecular function
0
Percent (%)

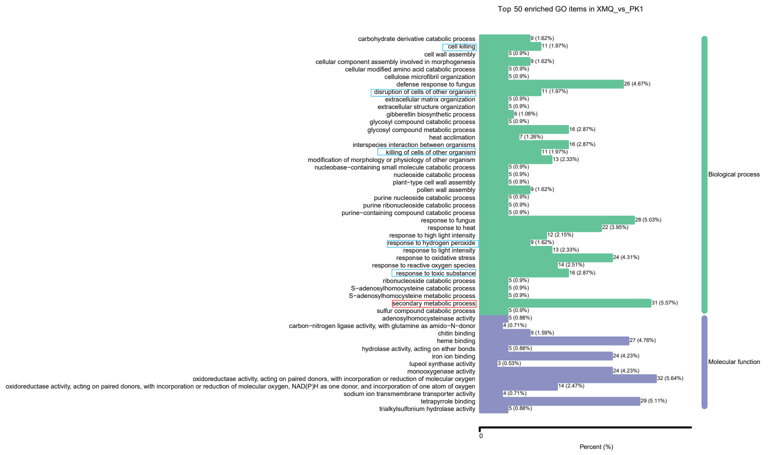

Top 50 enriched GO items in XMQ_vs_PK1
carbohydrate derivative catabolic process 9 (1.62%)
cell killing 11 (1.97%)
cell wall assembly 5 (0.9%)
cellular component assembly involved in morphogenesis 9 (1.62%)
cellular modified amino acid catabolic process 5 (0.9%)
cellulose microfibril organization 5 (0.9%)
defense response to fungus 26 (4.67%)
disruption of cells of other organism 11 (1.97%)
extracellular matrix organization 5 (0.9%)
extracellular structure organization 5 (0.9%)
gibberellin biosynthetic process 6 (1.08%)
glycosyl compound catabolic process 5 (0.9%)
glycosyl compound metabolic process 16 (2.87%)
heat acclimation 7 (1.26%)
interspecies interaction between organisms 16 (2.87%)
killing of cells of other organism 11 (1.97%)
modification of morphology or physiology of other organism 13 (2.33%)
nucleobase−containing small molecule catabolic process 5 (0.9%)
nucleoside catabolic process 5 (0.9%)
plant−type cell wall assembly 5 (0.9%)
pollen wall assembly 9 (1.62%)
purine nucleoside catabolic process 5 (0.9%)
purine ribonucleoside catabolic process 5 (0.9%)
purine−containing compound catabolic process 5 (0.9%)
response to fungus 28 (5.03%)
response to heat 22 (3.95%)
response to high light intensity 12 (2.15%)
response to hydrogen peroxide 9 (1.62%)
response to light intensity 13 (2.33%)
response to oxidative stress 24 (4.31%)
response to reactive oxygen species 14 (2.51%)
response to toxic substance 16 (2.87%)
ribonucleoside catabolic process 5 (0.9%)
S−adenosylhomocysteine catabolic process 5 (0.9%)
S−adenosylhomocysteine metabolic process 5 (0.9%)
secondary metabolic process 31 (5.57%)
sulfur compound catabolic process 5 (0.9%)
adenosylhomocysteinase activity 5 (0.88%)
carbon−nitrogen ligase activity, with glutamine as amido−N−donor 4 (0.71%)
chitin binding 9 (1.59%)
heme binding 27 (4.76%)
hydrolase activity, acting on ether bonds 5 (0.88%)
iron ion binding 24 (4.23%)
lupeol synthase activity 3 (0.53%)
monooxygenase activity 24 (4.23%)
oxidoreductase activity, acting on paired donors, with incorporation or reduction of molecular oxygen 32 (5.64%)
oxidoreductase activity, acting on paired donors, with incorporation or reduction of molecular oxygen, NAD(P)H as one donor, and incorporation of one atom of oxygen 14 (2.47%)
sodium ion transmembrane transporter activity 4 (0.71%)
tetrapyrrole binding 29 (5.11%)
trialkylsulfonium hydrolase activity 5 (0.88%)
Biological process
Molecular function
0
Percent (%)

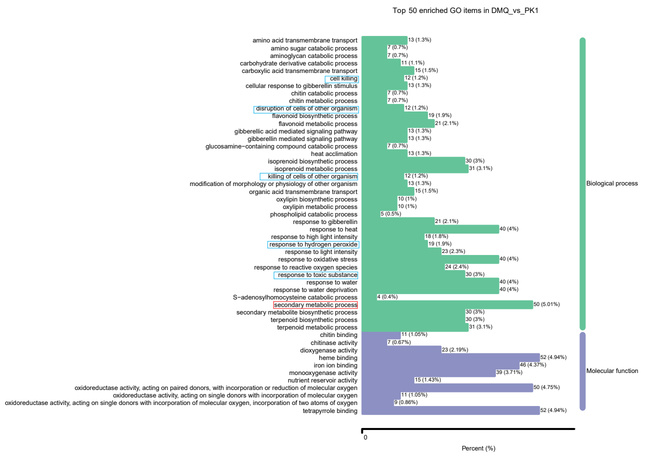

Top 50 enriched GO items in DMQ_vs_PK1
amino acid transmembrane transport 13 (1.3%)
amino sugar catabolic process 7 (0.7%)
aminoglycan catabolic process 7 (0.7%)
carbohydrate derivative catabolic process 11 (1.1%)
carboxylic acid transmembrane transport 15 (1.5%)
cell killing 12 (1.2%)
cellular response to gibberellin stimulus 13 (1.3%)
chitin catabolic process 7 (0.7%)
chitin metabolic process 7 (0.7%)
disruption of cells of other organism 12 (1.2%)
flavonoid biosynthetic process 19 (1.9%)
flavonoid metabolic process 21 (2.1%)
gibberellic acid mediated signaling pathway 13 (1.3%)
gibberellin mediated signaling pathway 13 (1.3%)
glucosamine−containing compound catabolic process 7 (0.7%)
heat acclimation 13 (1.3%)
isoprenoid biosynthetic process 30 (3%)
isoprenoid metabolic process 31 (3.1%)
killing of cells of other organism 12 (1.2%)
modification of morphology or physiology of other organism 13 (1.3%)
organic acid transmembrane transport 15 (1.5%)
oxylipin biosynthetic process 10 (1%)
oxylipin metabolic process 10 (1%)
phospholipid catabolic process 5 (0.5%)
response to gibberellin 21 (2.1%)
response to heat 40 (4%)
response to high light intensity 18 (1.8%)
response to hydrogen peroxide 19 (1.9%)
response to light intensity 23 (2.3%)
response to oxidative stress 40 (4%)
response to reactive oxygen species 24 (2.4%)
response to toxic substance 30 (3%)
response to water 40 (4%)
response to water deprivation 40 (4%)
S−adenosylhomocysteine catabolic process 4 (0.4%)
secondary metabolic process 50 (5.01%)
secondary metabolite biosynthetic process 30 (3%)
terpenoid biosynthetic process 30 (3%)
terpenoid metabolic process 31 (3.1%)
chitin binding 11 (1.05%)
chitinase activity 7 (0.67%)
dioxygenase activity 23 (2.19%)
heme binding 52 (4.94%)
iron ion binding 46 (4.37%)
monooxygenase activity 39 (3.71%)
nutrient reservoir activity 15 (1.43%)
oxidoreductase activity, acting on paired donors, with incorporation or reduction of molecular oxygen 50 (4.75%)
oxidoreductase activity, acting on single donors with incorporation of molecular oxygen 11 (1.05%)
oxidoreductase activity, acting on single donors with incorporation of molecular oxygen, incorporation of two atoms of oxygen 9 (0.86%)
tetrapyrrole binding 52 (4.94%)
Biological process
Molecular function
0
Percent (%)

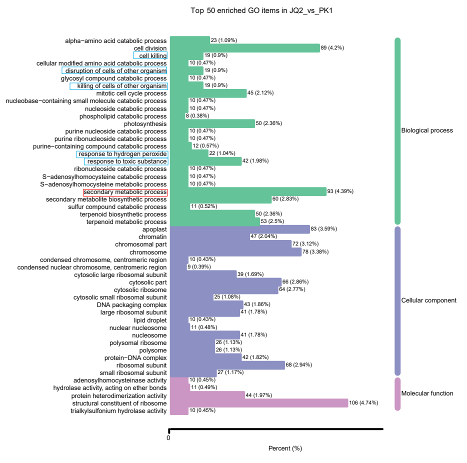

Top 50 enriched GO items in JQ2_vs_PK1
alpha−amino acid catabolic process 23 (1.09%)
cell division 89 (4.2%)
cell killing 19 (0.9%)
cellular modified amino acid catabolic process 10 (0.47%)
disruption of cells of other organism 19 (0.9%)
glycosyl compound catabolic process 10 (0.47%)
killing of cells of other organism 19 (0.9%)
mitotic cell cycle process 45 (2.12%)
nucleobase−containing small molecule catabolic process 10 (0.47%)
nucleoside catabolic process 10 (0.47%)
phospholipid catabolic process 8 (0.38%)
photosynthesis 50 (2.36%)
purine nucleoside catabolic process 10 (0.47%)
purine ribonucleoside catabolic process 10 (0.47%)
purine−containing compound catabolic process 12 (0.57%)
response to hydrogen peroxide 22 (1.04%)
response to toxic substance 42 (1.98%)
ribonucleoside catabolic process 10 (0.47%)
S−adenosylhomocysteine catabolic process 10 (0.47%)
S−adenosylhomocysteine metabolic process 10 (0.47%)
secondary metabolic process 93 (4.39%)
secondary metabolite biosynthetic process 60 (2.83%)
sulfur compound catabolic process 11 (0.52%)
terpenoid biosynthetic process 50 (2.36%)
terpenoid metabolic process 53 (2.5%)
apoplast 83 (3.59%)
chromatin 47 (2.04%)
chromosomal part 72 (3.12%)
chromosome 78 (3.38%)
condensed chromosome, centromeric region 10 (0.43%)
condensed nuclear chromosome, centromeric region 9 (0.39%)
cytosolic large ribosomal subunit 39 (1.69%)
cytosolic part 66 (2.86%)
cytosolic ribosome 64 (2.77%)
cytosolic small ribosomal subunit 25 (1.08%)
DNA packaging complex 43 (1.86%)
large ribosomal subunit 41 (1.78%)
lipid droplet 10 (0.43%)
nuclear nucleosome 11 (0.48%)
nucleosome 41 (1.78%)
polysomal ribosome 26 (1.13%)
polysome 26 (1.13%)
protein−DNA complex 42 (1.82%)
ribosomal subunit 68 (2.94%)
small ribosomal subunit 27 (1.17%)
adenosylhomocysteinase activity 10 (0.45%)
hydrolase activity, acting on ether bonds 11 (0.49%)
protein heterodimerization activity 44 (1.97%)
structural constituent of ribosome 106 (4.74%)
trialkylsulfonium hydrolase activity 10 (0.45%)
Biological process
Cellular component
Molecular function
0
Percent (%)

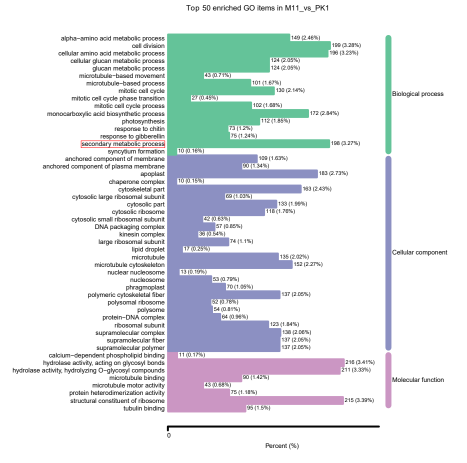

Top 50 enriched GO items in M11_vs_PK1
alpha−amino acid metabolic process 149 (2.46%)
cell division 199 (3.28%)
cellular amino acid metabolic process 196 (3.23%)
cellular glucan metabolic process 124 (2.05%)
glucan metabolic process 124 (2.05%)
microtubule−based movement 43 (0.71%)
microtubule−based process 101 (1.67%)
mitotic cell cycle 130 (2.14%)
mitotic cell cycle phase transition 27 (0.45%)
mitotic cell cycle process 102 (1.68%)
monocarboxylic acid biosynthetic process 172 (2.84%)
photosynthesis 112 (1.85%)
response to chitin 73 (1.2%)
response to gibberellin 75 (1.24%)
secondary metabolic process 198 (3.27%)
syncytium formation 10 (0.16%)
anchored component of membrane 109 (1.63%)
anchored component of plasma membrane 90 (1.34%)
apoplast 183 (2.73%)
chaperone complex 10 (0.15%)
cytoskeletal part 163 (2.43%)
cytosolic large ribosomal subunit 69 (1.03%)
cytosolic part 133 (1.99%)
cytosolic ribosome 118 (1.76%)
cytosolic small ribosomal subunit 42 (0.63%)
DNA packaging complex 57 (0.85%)
kinesin complex 36 (0.54%)
large ribosomal subunit 74 (1.1%)
lipid droplet 17 (0.25%)
microtubule 135 (2.02%)
microtubule cytoskeleton 152 (2.27%)
nuclear nucleosome 13 (0.19%)
nucleosome 53 (0.79%)
phragmoplast 70 (1.05%)
polymeric cytoskeletal fiber 137 (2.05%)
polysomal ribosome 52 (0.78%)
polysome 54 (0.81%)
protein−DNA complex 64 (0.96%)
ribosomal subunit 123 (1.84%)
supramolecular complex 138 (2.06%)
supramolecular fiber 137 (2.05%)
supramolecular polymer 137 (2.05%)
calcium−dependent phospholipid binding 11 (0.17%)
hydrolase activity, acting on glycosyl bonds 216 (3.41%)
hydrolase activity, hydrolyzing O−glycosyl compounds 211 (3.33%)
microtubule binding 90 (1.42%)
microtubule motor activity 43 (0.68%)
protein heterodimerization activity 75 (1.18%)
structural constituent of ribosome 215 (3.39%)
tubulin binding 95 (1.5%)
Biological process
Cellular component
Molecular function
0
Percent (%)
