# Supplementary figures and images for: Understanding the Potential Gene Regulatory Network of Starch Biosynthesis in Tartary Buckwheat by RNA-Seq
Source: Int J Mol Sci. 2022 Dec 12;23(24):15774. doi: 10.3390/ijms232415774 (PMC9779217; doi:10.3390/ijms232415774)

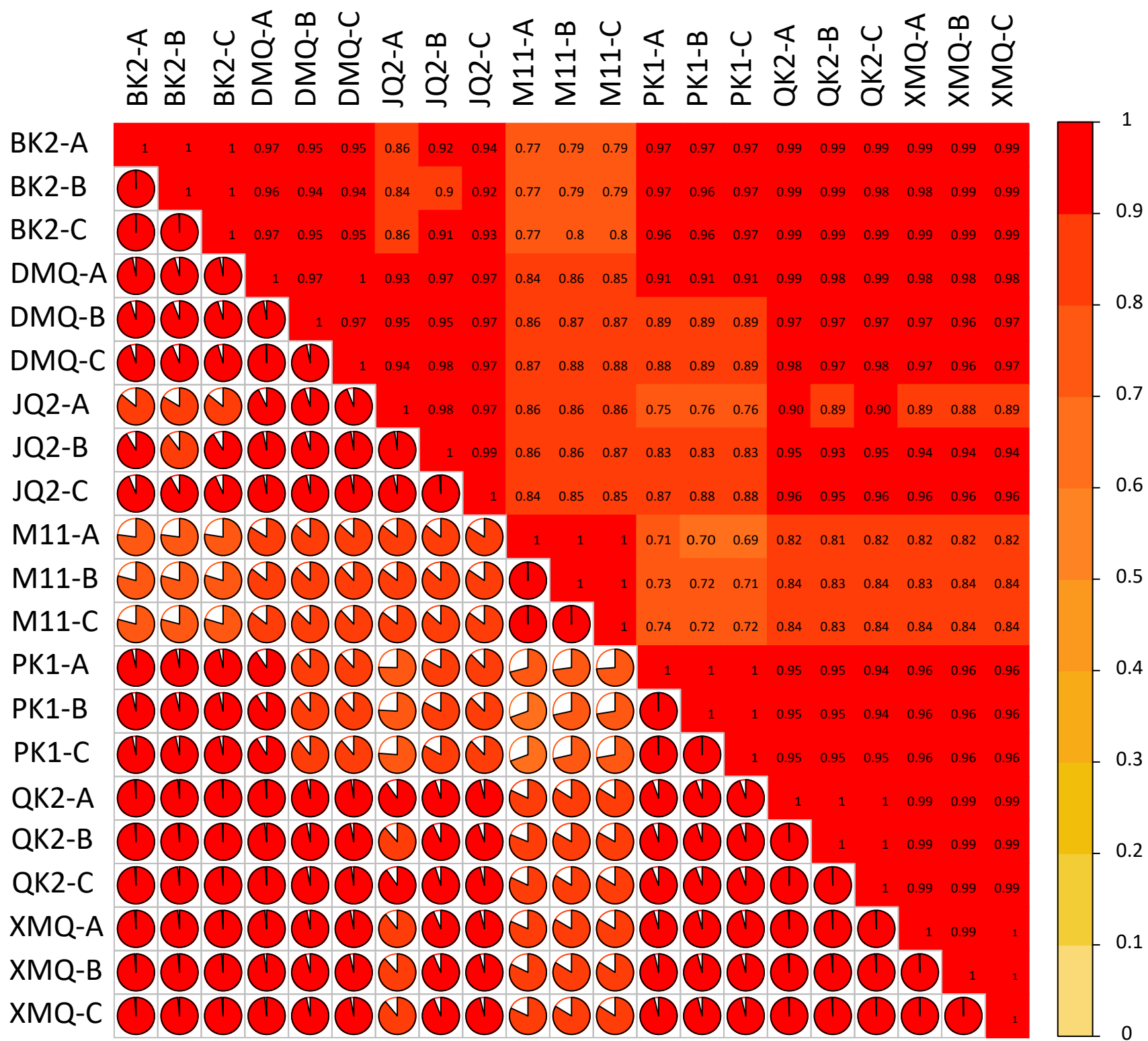

Supplement: Supplementary file 1 [file ijms-23-15774-s001.zip › Supplementary Figure S1. Sample cluster of the transcriptome. Each square indicates the Pear-soní»s correlation coefficient of a pair of samples..pdf]

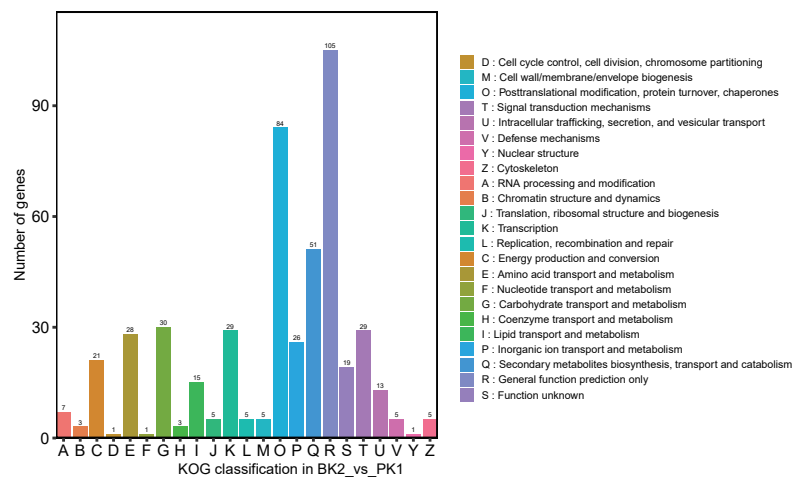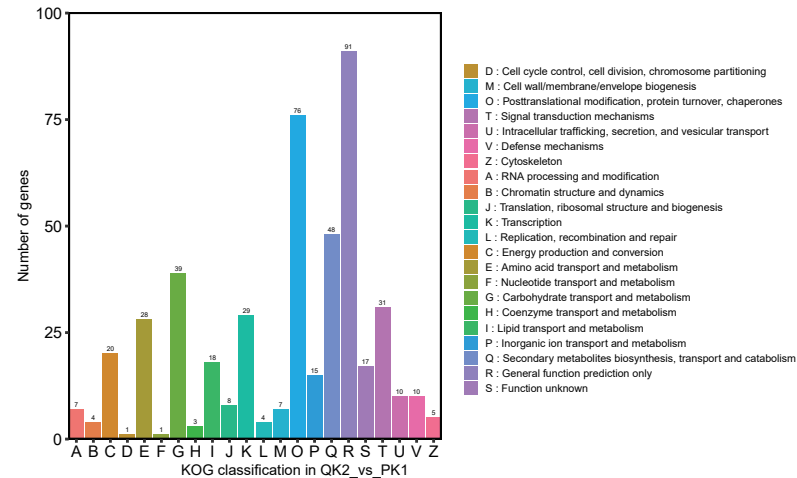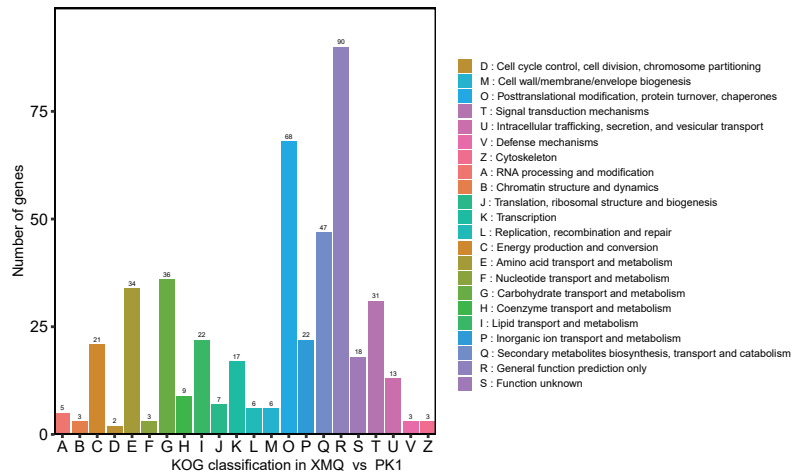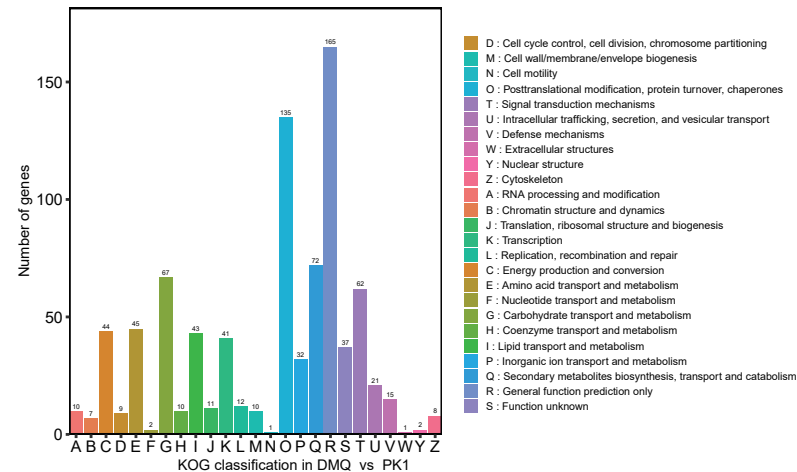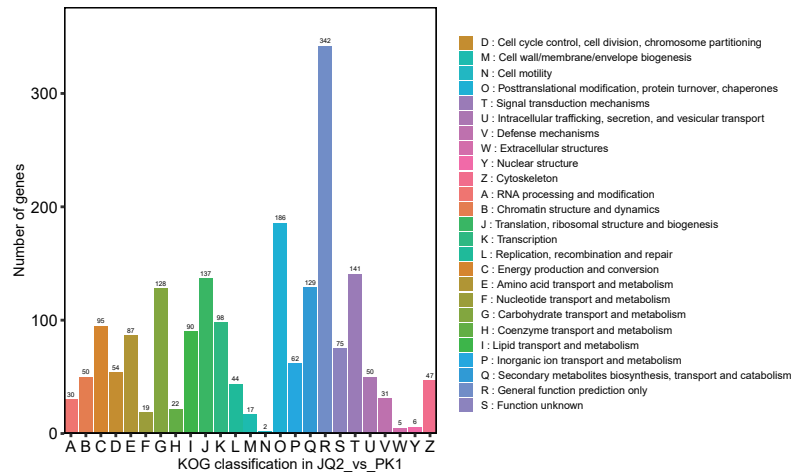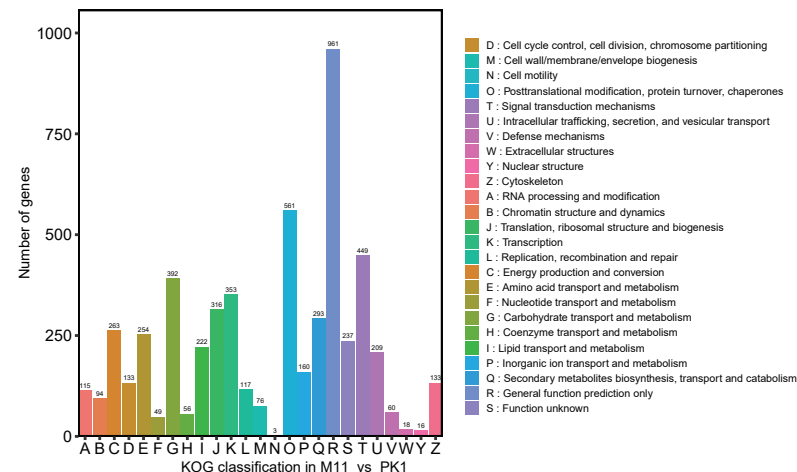

Supplement: Supplementary file 1 [file ijms-23-15774-s001.zip › Supplementary Figure S3. Histogram of the KOG (euKaryotic Ortholog Groups of proteins) clas-sification of the transcriptome.pdf]
